# An analysis of oral biopsies extracted from 1995 to 2009, in an oral medicine and surgery unit in Galicia (Spain)

**DOI:** 10.4317/medoral.17143

**Published:** 2011-07-15

**Authors:** Raquel Sixto-Requeijo, Marco Diniz-Freitas, Juan-Carlos Torreira-Lorenzo, Abel García-García, José M. Gándara-Rey

**Affiliations:** 1 Dentist. Assistant Professor of the Master of Oral Medicine, Surgery and Implantology of the Faculty of Dentistry of the University of Santiago de Compostela; 2 Professor of the Oral and Maxilofacial Unit. Faculty of Dentistry of the University of Santiago de Compostela. Head of the Maxilofacial Surgery Unit of the Santiago de Compostela Teaching Hospital; 3 Professor of the Oral and Maxilofacial Unit of the Faculty of Dentistry of the University of Santiago de Compostela

## Abstract

Objective: To conduct an analysis of the frequency of oral lesions in biopsies over a 14-year period in the Oral Medicine, Oral Surgery and Implantology Unit. Material and Methods: We conducted a retrospective study of biopsies removed from 1995-2009, recording data regarding age, sex, location of the lesions, biopsy types, anatomical and pathological diagnosis and definitive diagnosis. Results: Of the 562 patients studied, the average age was 51.8 years, with a standard deviation of 18.5 (range 5-96). The distribution by sex was 318 (56.6%) women and 244 (43.4%) men. The most common diagnostic category was mucosal pathologies in 37.9% of cases, followed by odontogenic cysts in 27.8%. Malignant tumors accounted for 3.9% of cases, oral squamous cell carcinomas were the most frequent malignancy, appearing in 22 cases. Bisphosphonate- related osteonecrosis of the jaws was the most common injury within the bone lesions group. Conclusion: Following the performance of 647 biopsies on 562 patients, we can say that the most common injury was radicular cysts (appearing in 108 cases), having found statistical differences in relation to the patients’ sex and age.

** Key words:** Frequency, oral pathology, biopsy.

## Introduction

Conducting an overall and detailed medical history and a comprehensive exploration of the oral cavity is essential to obtain correct diagnosis. Moreover this influences the prognosis and the implementation of the appropriate treatment for each patient. Thus we can detect existing lesions early, which is essential in malignancies and will guide the evolution and prognosis of the disease. Although occasionally it is possible to establish a clinical diagnosis, in most cases it is essential to conduct additional simple tests that provide valuable information, such as biopsies, which are a very useful diagnostic tool. In this paper we present the findings resulting of the analysis of 647 biopsies, performed over a 14-year period in the Master of Oral Medicine, Oral Surgery and Implantology at the University of Santiago de Compostela.

## Material and Methods

We conducted a retrospective study of biopsies removed from 1995-2009 at the Master of Oral Medicine, Oral Surgery and Im-plantology at the University of Santiago de Compostela. We reviewed the medical records of all patients undergoing the biopsies during this period, excluding cases in which we removed more than one biopsy from the same lesion and when any of the follow-ing data was missing: age, sex, location of lesions, type of biopsy, anatomical and pathological analysis and definitive diagnosis. Most patients were referred to our unit from different parts of Galicia, through the Galician Public Health Care Service (SERGAS) and private practices.

Lesions were then classified in 10 diagnostic categories, as detailed in ( Table 1). All results were subjected to statistical analysis using SPSS 12.0 for Windows Xp.

**Table 1:**
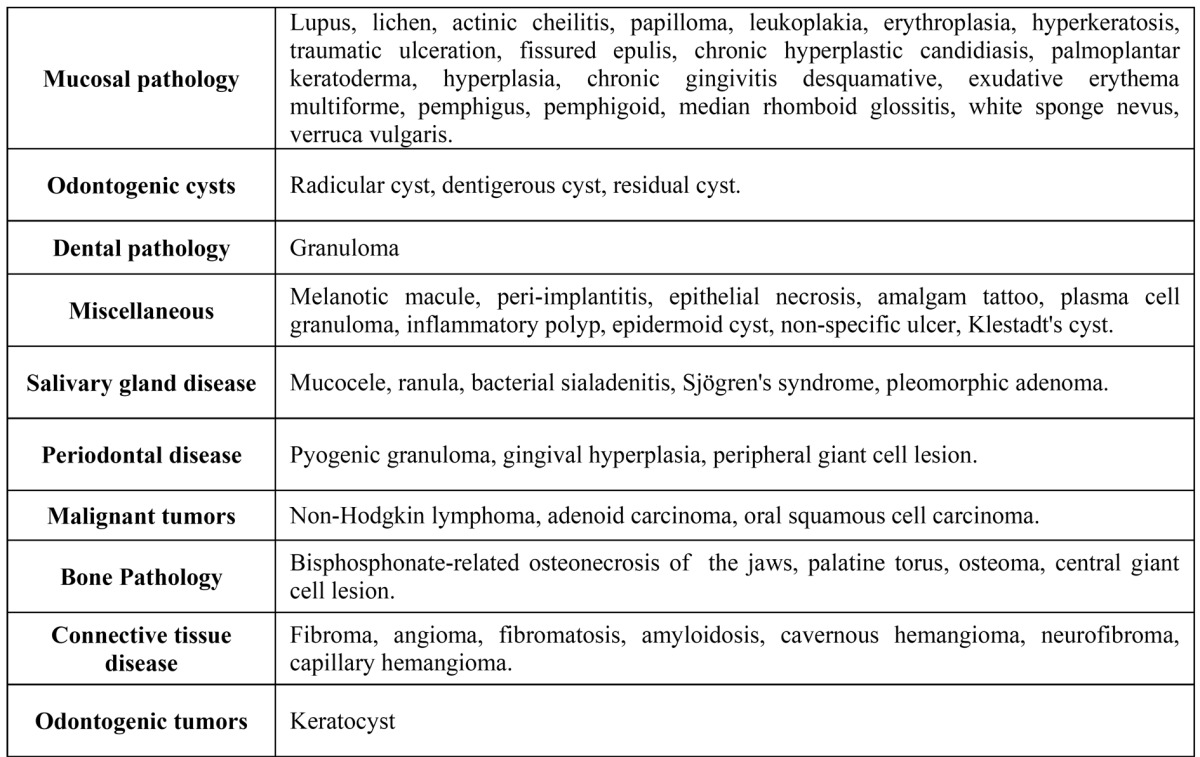
Classification of diagnoses by categories.

## Results

The number of patients studied was 562, which is less than the number of samples, indicating that in some cases more than one biopsy was taken. The average age of these patients was 51.8 with a standard deviation of 18.5 years (range 5-96). In terms of distribution according to sex 318 (56.6%) were women, whose average age was 51.2 ± 18.8 (standard deviation) and 244 (43.4%) men, whose average age was 50,1 ± 18.3 (standard deviation). The most common type of biopsy was excisional biopsy amounting to 66.5%. The most frequent lesion observed were radicular cysts, appearing in 108 cases (16.7%) followed by leukoplakia with 100 cases (15.5%), of which 15 showed different degrees of dysplasia in the histopathological study. The third most common lesion was lichen planus reaching 14.1%, followed by fibroma (11.4%). The prevalence of the diagnostic categories and their distribution with respect to sex can be seen in ( Table 2).

**Table 2:**
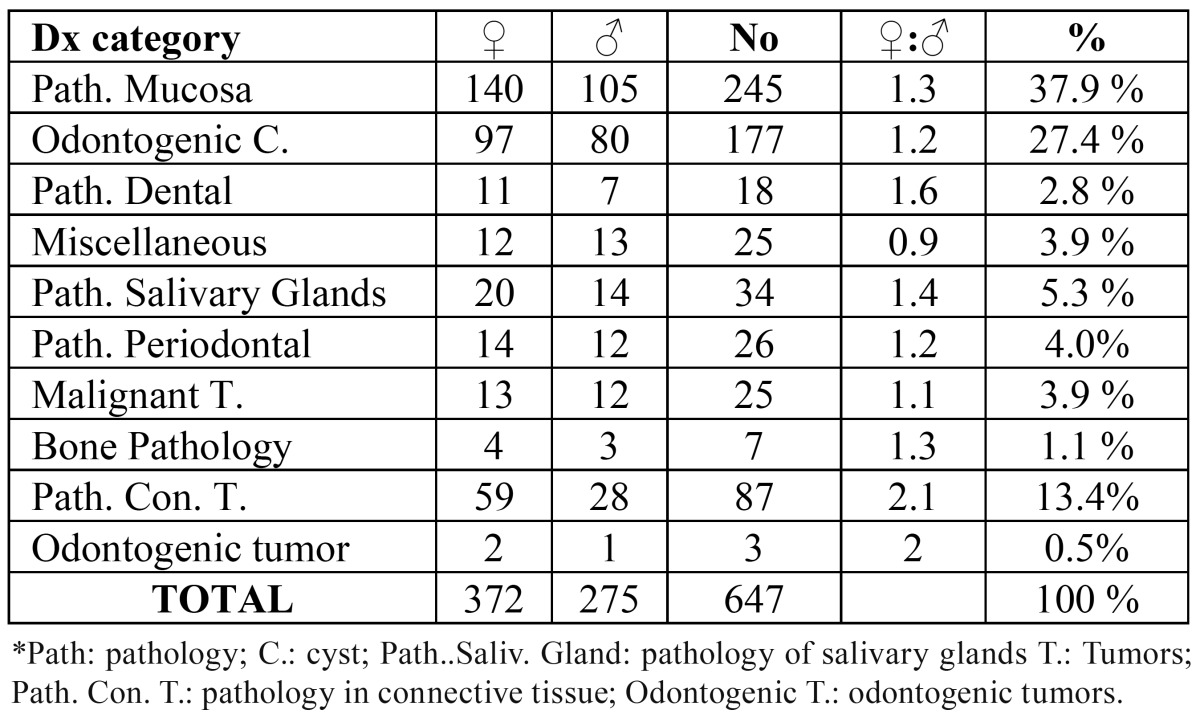
Diagnosis by category.

The most common injury in women was radicular cysts amounting to 65 cases; while in men leukoplakia appeared in 46 samples. The distribution of other lesions in relation to sex can be seen, distributed by diagnostic categories, in  Table 3,  Table 4.

**Table 3:**
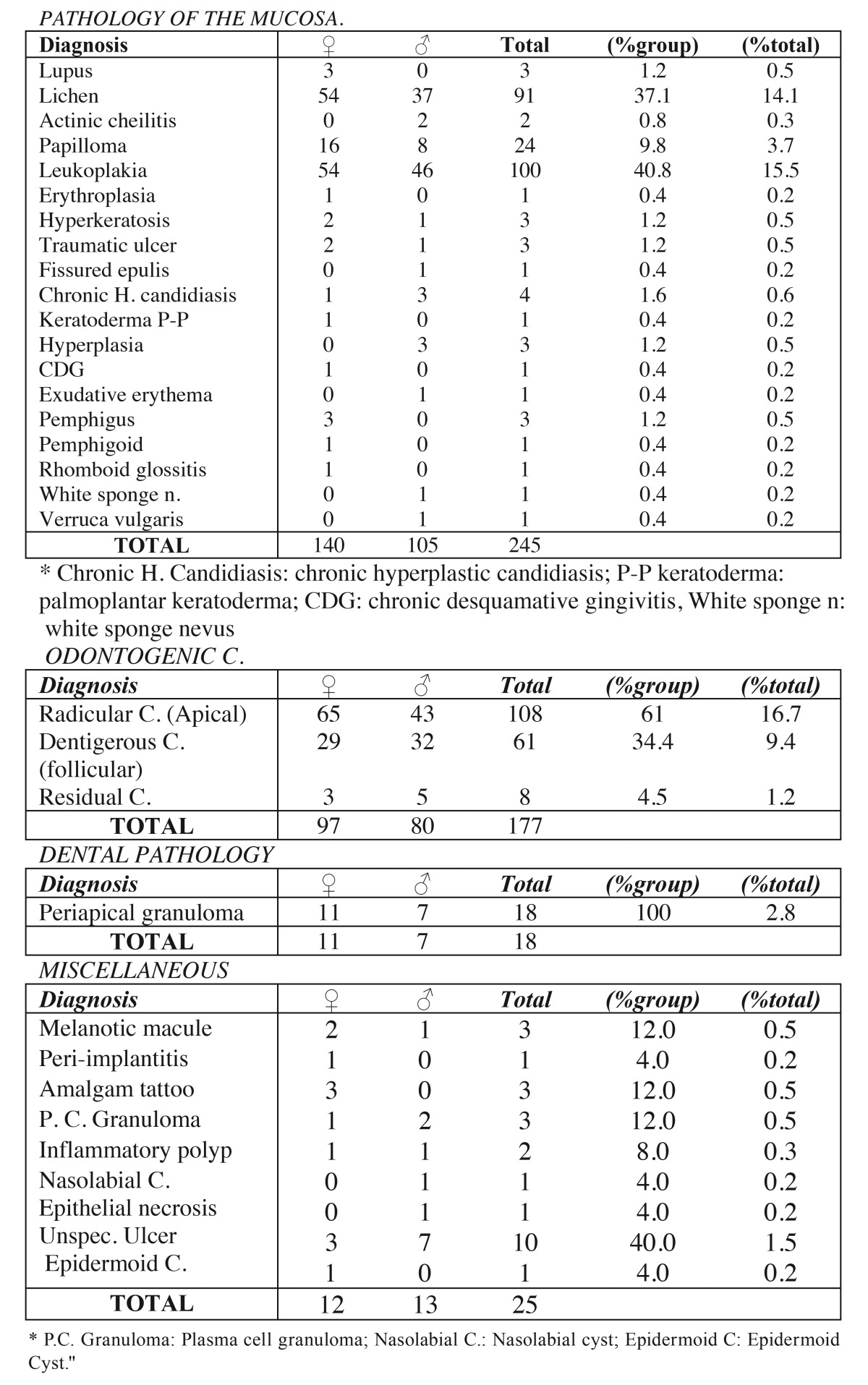
Contingency table. Sex and histological diagnosis (grouped by diagnostic categories).

**Table 4:**
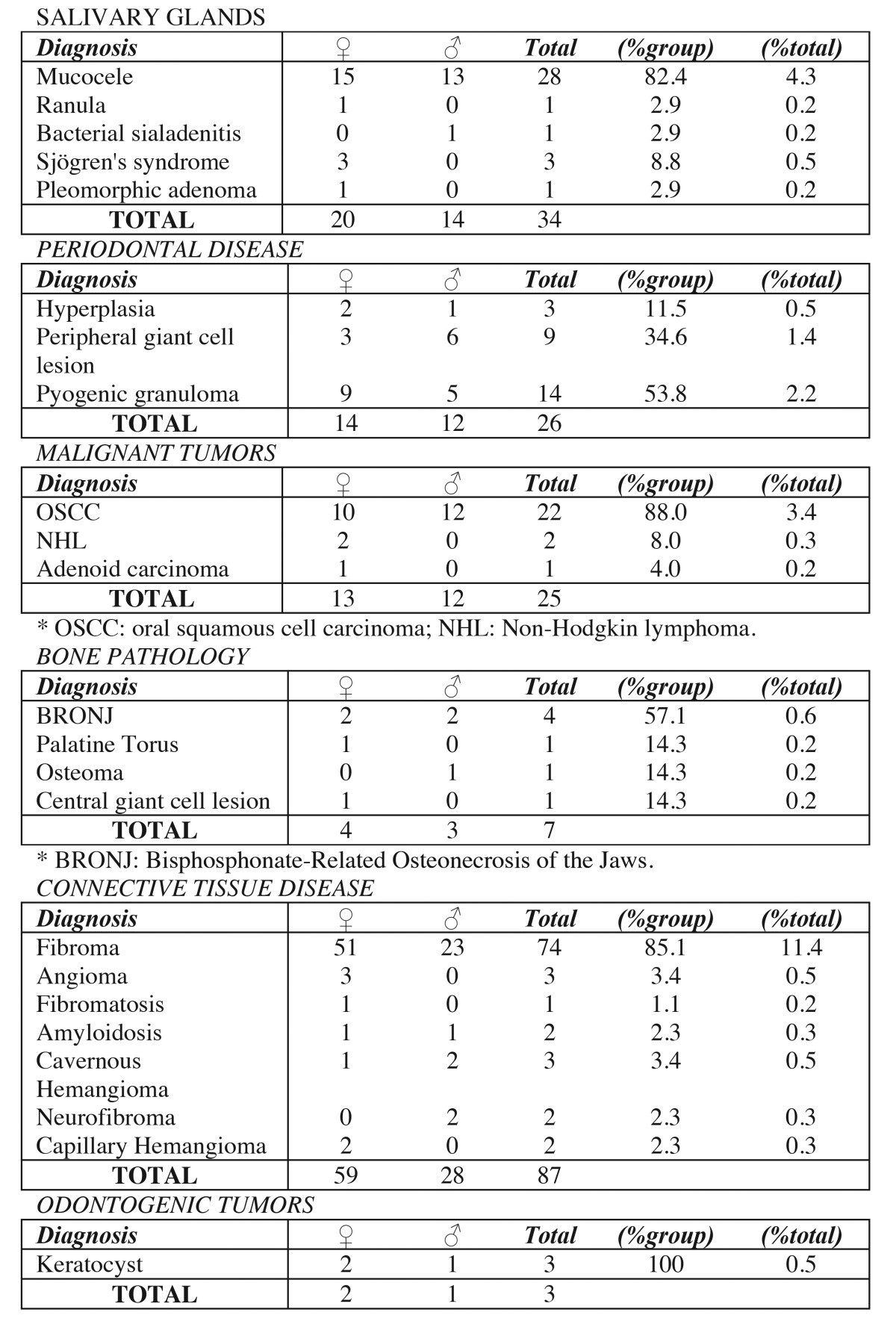
Contingency table. Sex and histological diagnosis (grouped by diagnostic categories)(cont).

Regarding the location, the most frequent injuries were those associated with a tooth, with a frequency of 23%, followed by gums (16.7%), tongue (15.9%), buccal mucosa (15.9%), lower lip (6%) and lesions associated with the third molar (5.9%), all other lesions amounted to less than 5%. The most frequent tooth injury was radicular cysts (apical); leukoplakia in the gum; lichen planus in buccal mucosa; leukoplakia in the tongue and mucocele in the lower lip area.

We divided the study population into 4 age groups, the results and most common diagnoses; as shown in ( Table 5).

**Table 5:**
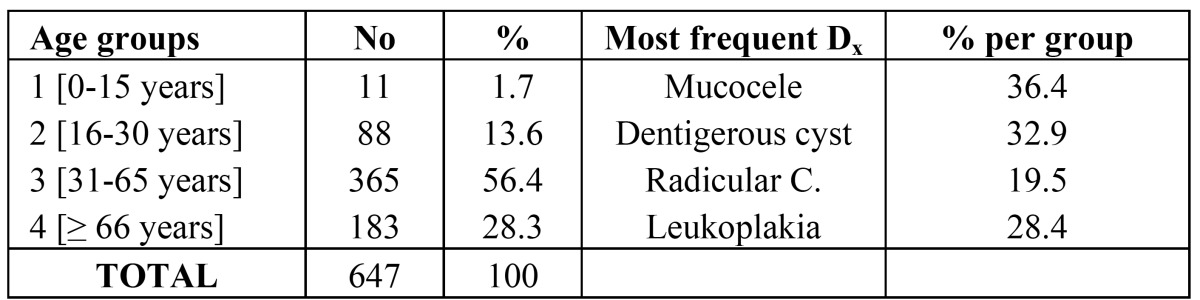
Prevalence of lesions according to age group.

## Discussion

In this study the number of lesions studied is much lower than those in other studies (1,2). Like other authors (1) we found a higher frequency of lesions in women, which can be easily explained since there are more women in our population, however, men showed a higher number of cases of actinic cheilitis and oral squamous cell carcinoma. The mean age was 51.8 years ± 18.5 (standard deviation) without excluding any group of children (3), therefore some results differ from those in other published studies, such as the study by Jones and Franklin (1), who only took into account patients over 17, or Das and Das (4) who studied injuries in children up to 20; and Shulman et al. (5), or Corrêa et al. (2) who focused their study on elderly patients (over 60). Nor have we focused on a particular condition or location as in other studies (6-8). 

By studying each diagnostic category separately, there are differences between our results and those of other authors (1) but these can be explained by changes made in classifying the various injuries in each diagnostic category. Given that our center is a specialized in oral medicine, thus a large number of cases with different mucosal diseases are referred to us from different parts of Galicia, both through the Galician Public Health Care Service (SERGAS) and through private practices, as mentioned above.

 Dental and periodontal pathologies

The most frequent dental pathology, as in other studies (1), was the periapical granuloma amounting to 2.8% of the total, a result lower than the one recorded by Jones and Franklin (1) who set it at 8.1% or Satorres et al. (9) (18.1%). Within the category of periodontal diseases pyogenic granuloma was the most common amounting to 53.8% (2.2% of total), a figure above those found in other studies (1) where the percentage was 31.8% within the group, but in our case this was due to the small sample size and the changes we made in classifying the lesions within the different diagnostic categories. The second most frequent diagnosis in this group was peripheral giant cell granuloma appearing in 9 cases (34.6%) (10).

 Odontogenic cysts 

With 27.8% of cases, this group stands second in frequency after pathology of the mucous membranes, figures higher than those recorded by other authors (14.9%) (11). Radicular cysts were the most prevalent lesion appearing in 108 cases, with a rate of 60% of all odontogenic cysts (16.7% of total). These results agree with other studies (11-17), which place periapical cysts as the most common odontogenic cysts, 39.9% (8), 50.7% (11), being the diagnosis showing the highest rates outside the odontogenic cysts’ group 19.5% (9), 41.2% (12). The second most common is the dentigerous cyst accounting for 33.9% of the group (9.4% of total) figures that are very similar to those found by Mosqueda et al. (33%) (8) but higher than those recorded by Tay (2.3%) (11); however, this may be due to sample size.

 Bone Pathology

In our study bisphosphonate-related osteonecrosis of the jaws was the largest lesion in this group accounting for 57.1% and 0.61% of the total. All cases were cancer patients treated with intravenous bisphosphonates to prevent bone metastases that had undergone dental extractions during treatment or suffered spontaneous loss of teeth. The data we found is not easily comparable, since similar studies are lacking biopsy data in cases diagnosed with bisphosphonates associated osteonecrosis (18-20). A rough estimate of this complication’s incidence could be drafted (namely, 0.8-12% in IV, and 0.01-0.04% in oral) (19); but further studies are required in the long term.

 Mucosal pathology 

Leukoplakia was the most common lesion in this group, 100 cases (40.8% of group and 15.5% of total), showing various degrees of dysplasia in 15 of them, followed by lichen planus (37.1% of the group, 14.1% of total), which were also among the most common injuries in other studies (1,2). All the cases were found in adults over age 30, in fact, research such as the one conducted by Corrêa et al. (2), who studied oral diseases in patients over 60 years, found that lichen planus reached a frequency of 61.29%.

 Connective tissue disease

Fibroma was the most common injury with 85.1%, representing 11.4% of the total. Its frequency was higher in women than in men of order 2.2:1, and the most common location were the buccal mucosa, gums and tongue. Torres et al. (6) in their study of 300 patients with benign tumors of the oral cavity found fibroma with a frequency of 53.3%, showing higher frequency in women than in men. However, these results are much higher than those reported by other authors (1,16,21). 

 Salivary glands disease

The most frequent injury was mucocele, which represented 4.3% of all injuries, appearing primarily among younger individuals (under 30). Although percentages vary between authors, 7.81% (17), 11.6% (4), 19.2% (22), it is considered the most frequent lesion of the salivary glands with a greater presence in children and young people who are particularly vulnerable, especially in the lower lip area, mainly due to its etiological association with traumatic factors (1,4,16).

 Malignant tumors

The group of malignant tumors accounted for 3.9%. Oral squamous cell carcinoma is considered the most common malignancy of the oral cavity appearing mostly on the tongue and lower lip. In our study it amounted to 88% of all malignant lesions (3.4% of total), the most common location was the tongue (14 cases), and its appearance was higher in men and in patients belonging to the fourth age group (≥ 66 years). Jones and Franklin (1) reported that oral squamous cell carcinoma was the most common malignancy in their study (66.1%) with a higher prevalence in men, with an average age of 64.2 years. Mujica et al. (3) found that oral squamous cell carcinomas showed frequency of 2% among 306 injuries, while Satorres et al. recorded 1% (9) and Skinner and Weir (23) reported 5.4% in 2675 samples, but this higher percentage is due to the fact that their study population was older than 55.

 Odontogenic tumors

Although works, such as the one by Jones and Franklin (1), account for a larger number of injuries within this group, however, we have only found keratocysts, showing 0.5% frequency over the total; these figures are similar to those published in other papers 0.01% (1) 0.8% (23).

Ultimately, we can conclude that radicular cysts were the most common lesion found in our work, with 108 cases and a higher prevalence in adults (16-65 years). Likewise, the percentage of injuries was higher in women, although the frequency of premalignant and malignant lesions was higher in men over age 50.
